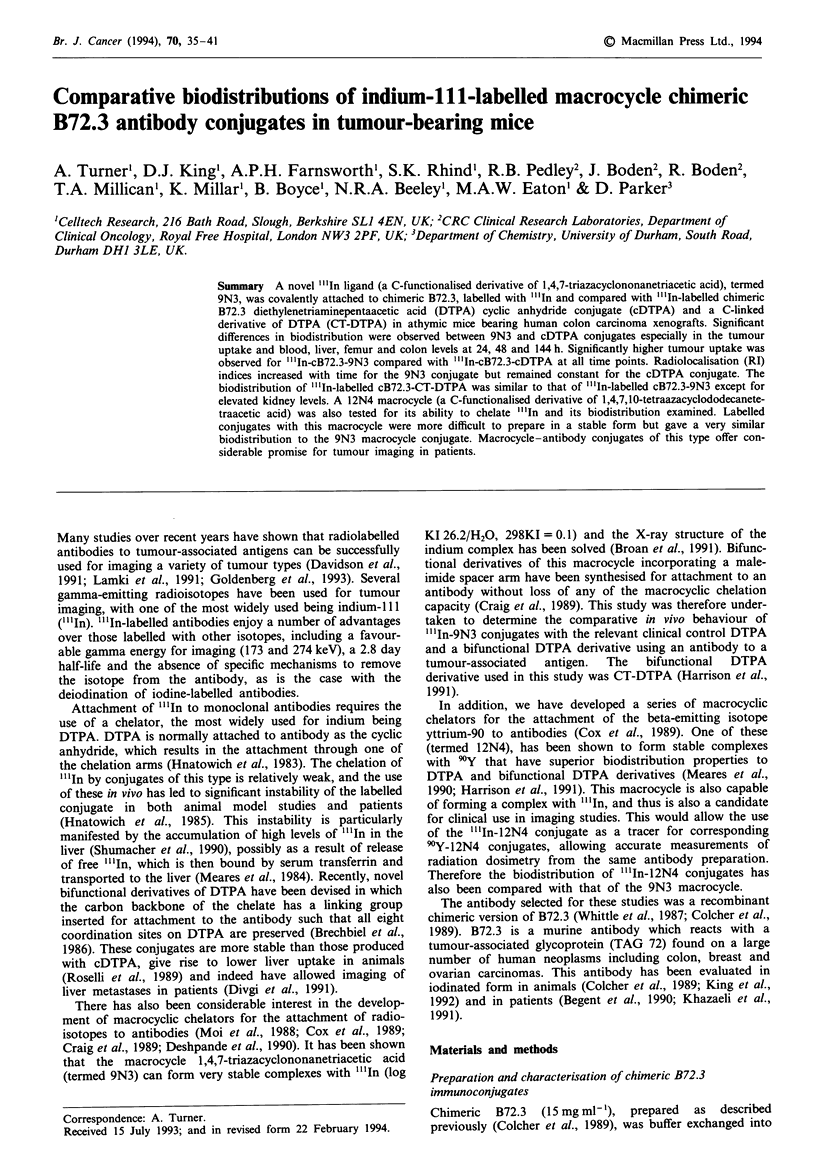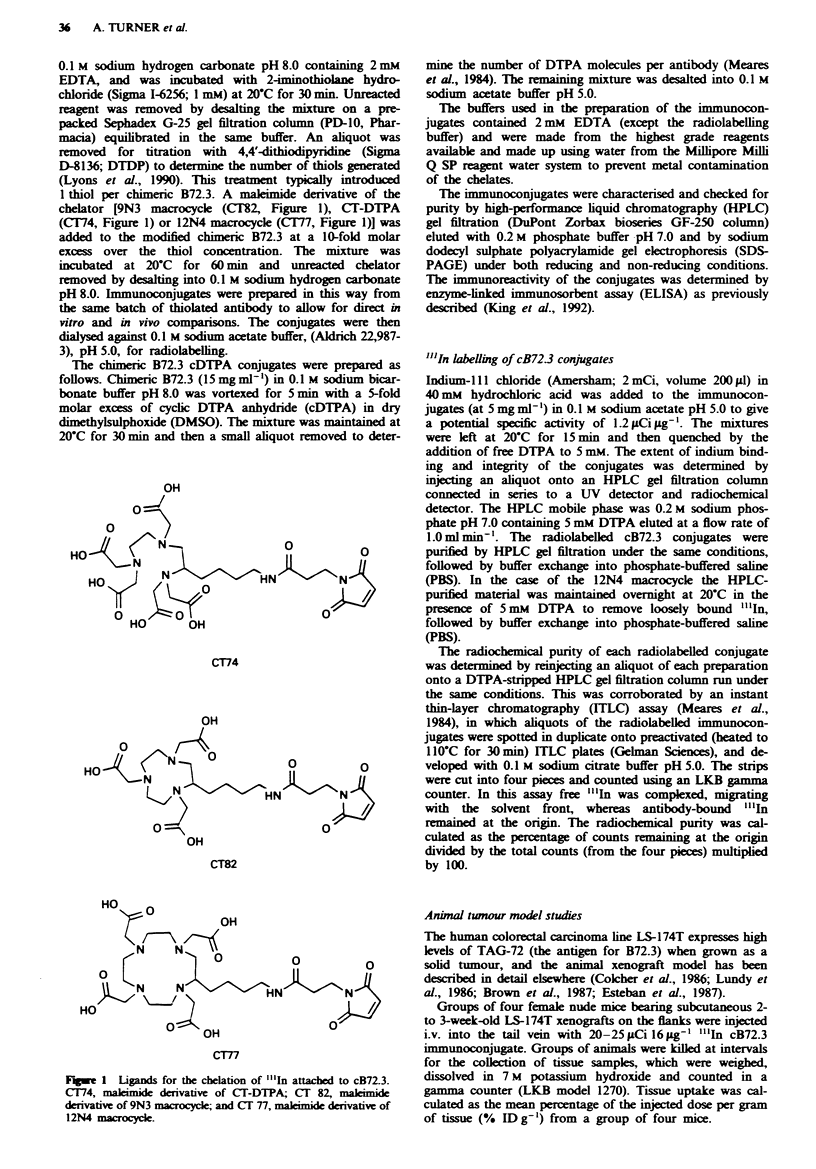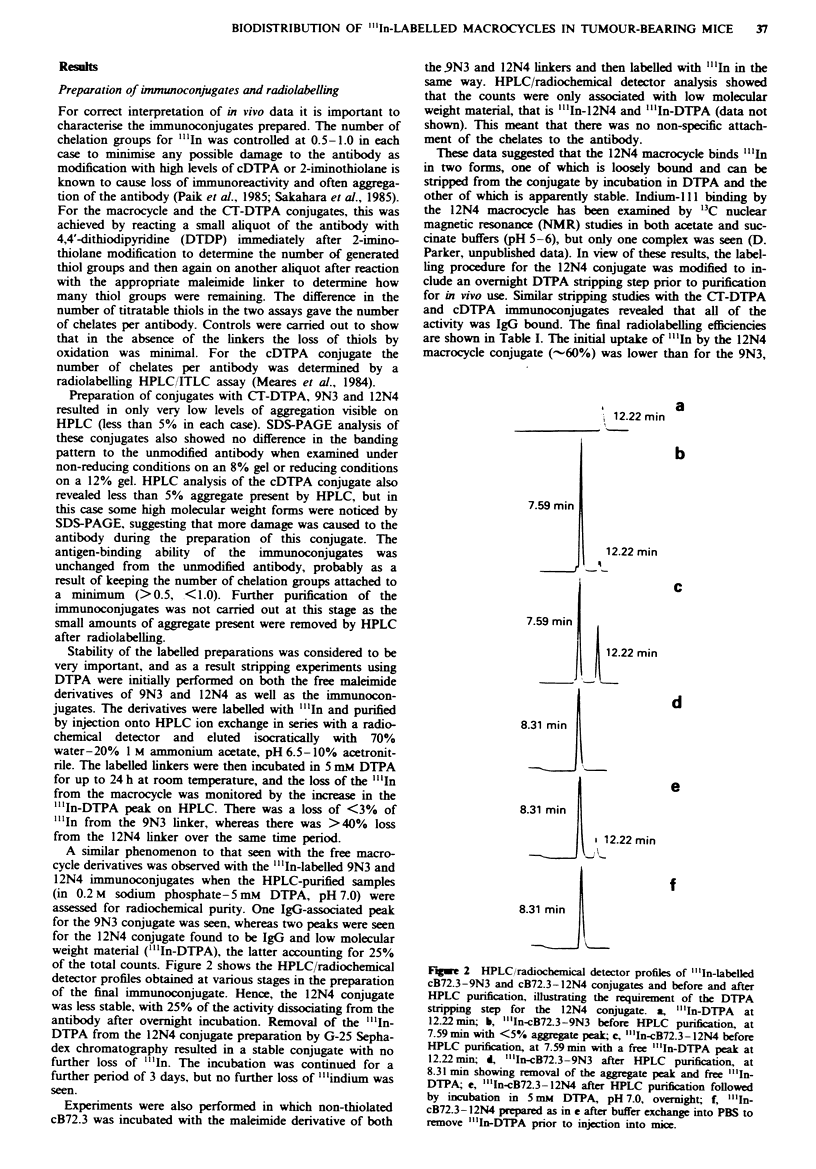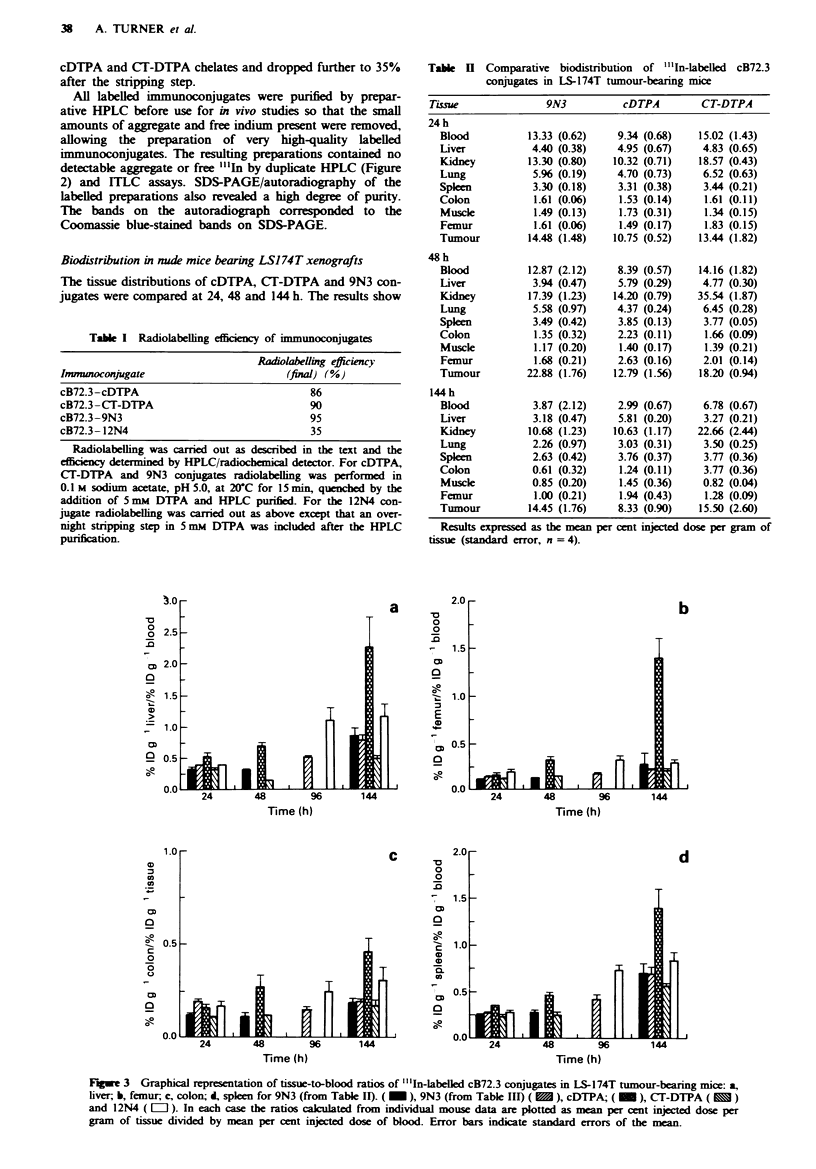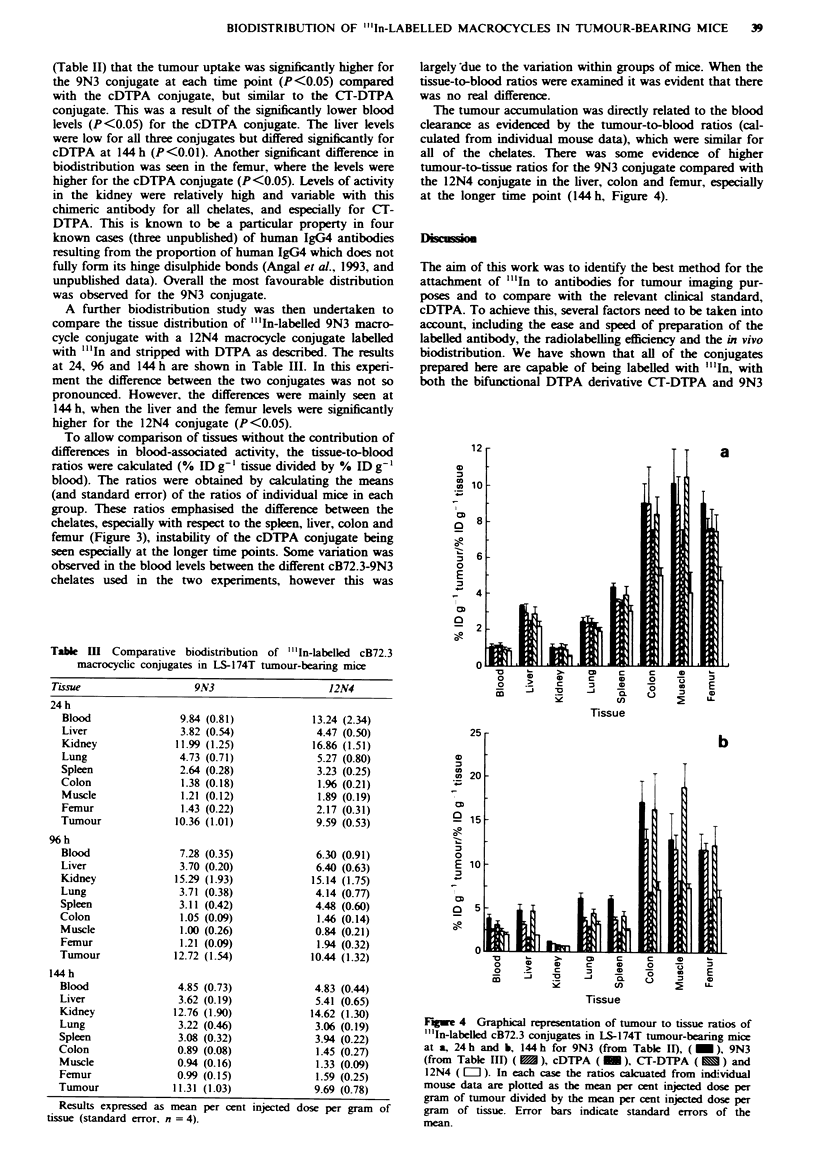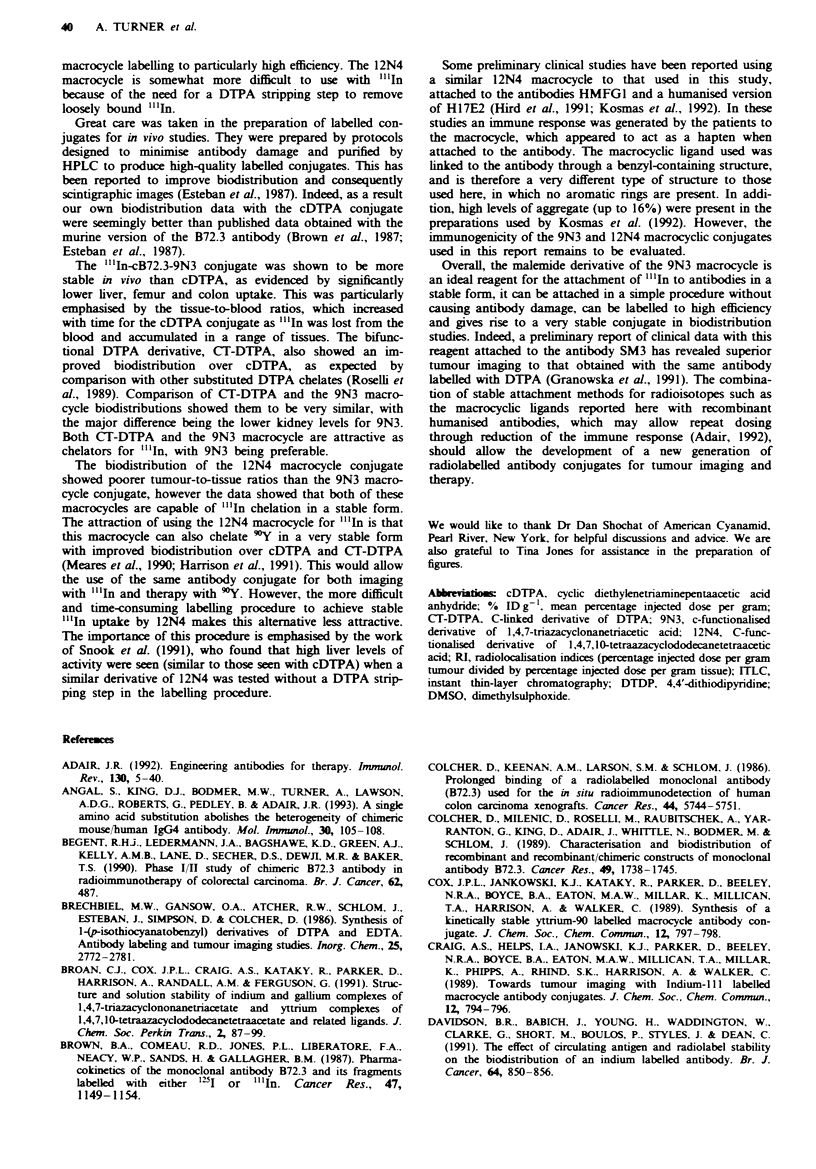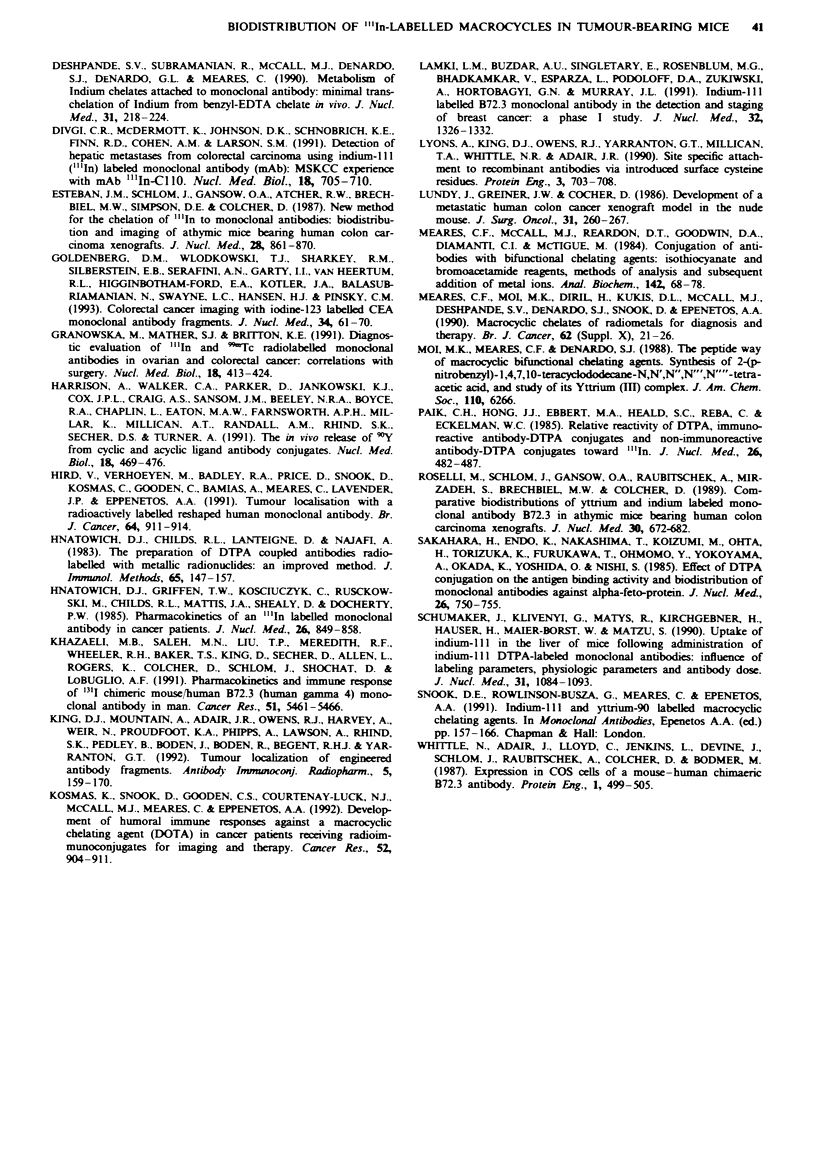# Comparative biodistributions of indium-111-labelled macrocycle chimeric B72.3 antibody conjugates in tumour-bearing mice.

**DOI:** 10.1038/bjc.1994.246

**Published:** 1994-07

**Authors:** A. Turner, D. J. King, A. P. Farnsworth, S. K. Rhind, R. B. Pedley, J. Boden, R. Boden, T. A. Millican, K. Millar, B. Boyce

**Affiliations:** Celltech Research, Slough, Berkshire, UK.

## Abstract

A novel 111In ligand (a C-functionalised derivative of 1,4,7-triazacyclononanetriacetic acid), termed 9N3, was covalently attached to chimeric B72.3, labelled with 111In and compared with 111In-labelled chimeric B72.3 diethylenetriaminepentaacetic acid (DTPA) cyclic anhydride conjugate (cDTPA) and a C-linked derivative of DTPA (CT-DTPA) in athymic mice bearing human colon carcinoma xenografts. Significant differences in biodistribution were observed between 9N3 and cDTPA conjugates especially in the tumour uptake and blood, liver, femur and colon levels at 24, 48 and 144 h. Significantly higher tumour uptake was observed for 111In-cB72.3-9N3 compared with 111In-cB72.3-cDTPA at all time points. Radiolocalisation (RI) indices increased with time for the 9N3 conjugate but remained constant for the cDTPA conjugate. The biodistribution of 111In-labelled cB72.3-CT-DTPA was similar to that of 111In-labelled cB72.3-9N3 except for elevated kidney levels. A 12N4 macrocycle (a C-functionalised derivative of 1,4,7,10-tetraazacyclododecanetetraacetic acid) was also tested for its ability to chelate 111In and its biodistribution examined. Labelled conjugates with this macrocycle were more difficult to prepare in a stable form but gave a very similar biodistribution to the 9N3 macrocycle conjugate. Macrocycle-antibody conjugates of this type offer considerable promise for tumour imaging in patients.


*Br. J. Cancer* (1994), **70**, 35–41 © Macmillan Press Ltd., 1994

### Comparative biodistributions of indium-111-labelled macrocycle chimeric B72.3 antibody conjugates in tumour-bearing mice

A. Turner[1], D.J. King[1], A.P.H. Farnsworth[1], S.K. Rhind[1], R.B. Pedley[2], J. Boden[2], R. Boden[2], T.A. Millican[1], K. Millar[1], B. Boyce[1], N.R.A. Beeley[1], M.A.W. Eaton[1] & D. Parker[3]

*[1]Celltech Research, 216 Bath Road, Slough, Berkshire SL1 4EN, UK; [2]CRC Clinical Research Laboratories, Department of Clinical Oncology, Royal Free Hospital, London NW3 2PF, UK; [3]Department of Chemistry, University of Durham, South Road, Durham DH1 3LE, UK.*

**Summary** A novel $^{111}$In ligand (a C-functionalised derivative of 1,4,7-triazacyclononanetriacetic acid), termed 9N3, was covalently attached to chimeric B72.3, labelled with $^{111}$In and compared with $^{111}$In-labelled chimeric B72.3 diethylenetriaminepentaacetic acid (DTPA) cyclic anhydride conjugate (cDTPA) and a C-linked derivative of DTPA (CT-DTPA) in athymic mice bearing human colon carcinoma xenografts. Significant differences in biodistribution were observed between 9N3 and cDTPA conjugates especially in the tumour uptake and blood, liver, femur and colon levels at 24, 48 and 144 h. Significantly higher tumour uptake was observed for $^{111}$In-cB72.3-9N3 compared with $^{111}$In-cB72.3-cDTPA at all time points. Radiolocalisation (RI) indices increased with time for the 9N3 conjugate but remained constant for the cDTPA conjugate. The biodistribution of $^{111}$In-labelled cB72.3-CT-DTPA was similar to that of $^{111}$In-labelled cB72.3-9N3 except for elevated kidney levels. A 12N4 macrocycle (a C-functionalised derivative of 1,4,7,10-tetraazacyclododecanetetraacetic acid) was also tested for its ability to chelate $^{111}$In and its biodistribution examined. Labelled conjugates with this macrocycle were more difficult to prepare in a stable form but gave a very similar biodistribution to the 9N3 macrocycle conjugate. Macrocycle–antibody conjugates of this type offer considerable promise for tumour imaging in patients.

Many studies over recent years have shown that radiolabelled antibodies to tumour-associated antigens can be successfully used for imaging a variety of tumour types (Davidson *et al.*, 1991; Lamki *et al.*, 1991; Goldenberg *et al.*, 1993). Several gamma-emitting radioisotopes have been used for tumour imaging, with one of the most widely used being indium-111 ($^{111}$In). $^{111}$In-labelled antibodies enjoy a number of advantages over those labelled with other isotopes, including a favourable gamma energy for imaging (173 and 274 keV), a 2.8 day half-life and the absence of specific mechanisms to remove the isotope from the antibody, as is the case with the deiodination of iodine-labelled antibodies.

Attachment of $^{111}$In to monoclonal antibodies requires the use of a chelator, the most widely used for indium being DTPA. DTPA is normally attached to antibody as the cyclic anhydride, which results in the attachment through one of the chelation arms (Hnatowich *et al.*, 1983). The chelation of $^{111}$In by conjugates of this type is relatively weak, and the use of these *in vivo* has led to significant instability of the labelled conjugate in both animal model studies and patients (Hnatowich *et al.*, 1985). This instability is particularly manifested by the accumulation of high levels of $^{111}$In in the liver (Shumacher *et al.*, 1990), possibly as a result of release of free $^{111}$In, which is then bound by serum transferrin and transported to the liver (Meares *et al.*, 1984). Recently, novel bifunctional derivatives of DTPA have been devised in which the carbon backbone of the chelate has a linking group inserted for attachment to the antibody such that all eight coordination sites on DTPA are preserved (Brechbiel *et al.*, 1986). These conjugates are more stable than those produced with cDTPA, give rise to lower liver uptake in animals (Roselli *et al.*, 1989) and indeed have allowed imaging of liver metastases in patients (Divgi *et al.*, 1991).

There has also been considerable interest in the development of macrocyclic chelators for the attachment of radioisotopes to antibodies (Moi *et al.*, 1988; Cox *et al.*, 1989; Craig *et al.*, 1989; Deshpande *et al.*, 1990). It has been shown that the macrocycle 1,4,7-triazacyclononanetriacetic acid (termed 9N3) can form very stable complexes with $^{111}$In (log KI 26.2/$H_2O$, 298KI = 0.1) and the X-ray structure of the indium complex has been solved (Broan *et al.*, 1991). Bifunctional derivatives of this macrocycle incorporating a maleimide spacer arm have been synthesised for attachment to an antibody without loss of any of the macrocyclic chelation capacity (Craig *et al.*, 1989). This study was therefore undertaken to determine the comparative *in vivo* behaviour of $^{111}$In-9N3 conjugates with the relevant clinical control DTPA and a bifunctional DTPA derivative using an antibody to a tumour-associated antigen. The bifunctional DTPA derivative used in this study was CT-DTPA (Harrison *et al.*, 1991).

In addition, we have developed a series of macrocyclic chelators for the attachment of the beta-emitting isotope yttrium-90 to antibodies (Cox *et al.*, 1989). One of these (termed 12N4), has been shown to form stable complexes with $^{90}$Y that have superior biodistribution properties to DTPA and bifunctional DTPA derivatives (Meares *et al.*, 1990; Harrison *et al.*, 1991). This macrocycle is also capable of forming a complex with $^{111}$In, and thus is also a candidate for clinical use in imaging studies. This would allow the use of the $^{111}$In-12N4 conjugate as a tracer for corresponding $^{90}$Y-12N4 conjugates, allowing accurate measurements of radiation dosimetry from the same antibody preparation. Therefore the biodistribution of $^{111}$In-12N4 conjugates has also been compared with that of the 9N3 macrocycle.

The antibody selected for these studies was a recombinant chimeric version of B72.3 (Whittle *et al.*, 1987; Colcher *et al.*, 1989). B72.3 is a murine antibody which reacts with a tumour-associated glycoprotein (TAG 72) found on a large number of human neoplasms including colon, breast and ovarian carcinomas. This antibody has been evaluated in iodinated form in animals (Colcher *et al.*, 1989; King *et al.*, 1992) and in patients (Begent *et al.*, 1990; Khazaeli *et al.*, 1991).

### Materials and methods

### *Preparation and characterisation of chimeric B72.3 immunoconjugates*

Chimeric B72.3 (15 mg ml$^{-1}$), prepared as described previously (Colcher *et al.*, 1989), was buffer exchanged into

Correspondence: A. Turner.
Received 15 July 1993; and in revised form 22 February 1994.

0.1 M sodium hydrogen carbonate pH 8.0 containing 2 mM EDTA, and was incubated with 2-iminothiolane hydrochloride (Sigma I-6256; 1 mM) at 20°C for 30 min. Unreacted reagent was removed by desalting the mixture on a prepacked Sephadex G-25 gel filtration column (PD-10, Pharmacia) equilibrated in the same buffer. An aliquot was removed for titration with 4,4'-dithiodipyridine (Sigma D-8136; DTDP) to determine the number of thiols generated (Lyons et al., 1990). This treatment typically introduced 1 thiol per chimeric B72.3. A maleimide derivative of the chelator [9N3 macrocycle (CT82, Figure 1), CT-DTPA (CT74, Figure 1) or 12N4 macrocycle (CT77, Figure 1)] was added to the modified chimeric B72.3 at a 10-fold molar excess over the thiol concentration. The mixture was incubated at 20°C for 60 min and unreacted chelator removed by desalting into 0.1 M sodium hydrogen carbonate pH 8.0. Immunoconjugates were prepared in this way from the same batch of thiolated antibody to allow for direct *in vitro* and *in vivo* comparisons. The conjugates were then dialysed against 0.1 M sodium acetate buffer, (Aldrich 22,987-3), pH 5.0, for radiolabelling.

The chimeric B72.3 cDTPA conjugates were prepared as follows. Chimeric B72.3 (15 mg $ml^{-1}$) in 0.1 M sodium bicarbonate buffer pH 8.0 was vortexed for 5 min with a 5-fold molar excess of cyclic DTPA anhydride (cDTPA) in dry dimethylsulphoxide (DMSO). The mixture was maintained at 20°C for 30 min and then a small aliquot removed to determine the number of DTPA molecules per antibody (Meares et al., 1984). The remaining mixture was desalted into 0.1 M sodium acetate buffer pH 5.0.

CT74

CT82

CT77

**Figure 1** Ligands for the chelation of $^{111}$In attached to cB72.3. CT74, maleimide derivative of CT-DTPA; CT 82, maleimide derivative of 9N3 macrocycle; and CT 77, maleimide derivative of 12N4 macrocycle.

The buffers used in the preparation of the immunoconjugates contained 2 mM EDTA (except the radiolabelling buffer) and were made from the highest grade reagents available and made up using water from the Millipore Milli Q SP reagent water system to prevent metal contamination of the chelates.

The immunoconjugates were characterised and checked for purity by high-performance liquid chromatography (HPLC) gel filtration (DuPont Zorbax bioseries GF-250 column) eluted with 0.2 M phosphate buffer pH 7.0 and by sodium dodecyl sulphate polyacrylamide gel electrophoresis (SDS-PAGE) under both reducing and non-reducing conditions. The immunoreactivity of the conjugates was determined by enzyme-linked immunosorbent assay (ELISA) as previously described (King et al., 1992).

### *$^{111}$In labelling of cB72.3 conjugates*

Indium-111 chloride (Amersham; 2 mCi, volume 200 µl) in 40 mM hydrochloric acid was added to the immunoconjugates (at 5 mg $ml^{-1}$) in 0.1 M sodium acetate pH 5.0 to give a potential specific activity of 1.2 µCi $\mu g^{-1}$. The mixtures were left at 20°C for 15 min and then quenched by the addition of free DTPA to 5 mM. The extent of indium binding and integrity of the conjugates was determined by injecting an aliquot onto an HPLC gel filtration column connected in series to a UV detector and radiochemical detector. The HPLC mobile phase was 0.2 M sodium phosphate pH 7.0 containing 5 mM DTPA eluted at a flow rate of 1.0 ml $min^{-1}$. The radiolabelled cB72.3 conjugates were purified by HPLC gel filtration under the same conditions, followed by buffer exchange into phosphate-buffered saline (PBS). In the case of the 12N4 macrocycle the HPLC-purified material was maintained overnight at 20°C in the presence of 5 mM DTPA to remove loosely bound $^{111}$In, followed by buffer exchange into phosphate-buffered saline (PBS).

The radiochemical purity of each radiolabelled conjugate was determined by reinjecting an aliquot of each preparation onto a DTPA-stripped HPLC gel filtration column run under the same conditions. This was corroborated by an instant thin-layer chromatography (ITLC) assay (Meares et al., 1984), in which aliquots of the radiolabelled immunoconjugates were spotted in duplicate onto preactivated (heated to 110°C for 30 min) ITLC plates (Gelman Sciences), and developed with 0.1 M sodium citrate buffer pH 5.0. The strips were cut into four pieces and counted using an LKB gamma counter. In this assay free $^{111}$In was complexed, migrating with the solvent front, whereas antibody-bound $^{111}$In remained at the origin. The radiochemical purity was calculated as the percentage of counts remaining at the origin divided by the total counts (from the four pieces) multiplied by 100.

### *Animal tumour model studies*

The human colorectal carcinoma line LS-174T expresses high levels of TAG-72 (the antigen for B72.3) when grown as a solid tumour, and the animal xenograft model has been described in detail elsewhere (Colcher et al., 1986; Lundy et al., 1986; Brown et al., 1987; Esteban et al., 1987).

Groups of four female nude mice bearing subcutaneous 2- to 3-week-old LS-174T xenografts on the flanks were injected i.v. into the tail vein with 20–25 µCi 16 $\mu g^{-1}$ $^{111}$In cB72.3 immunoconjugate. Groups of animals were killed at intervals for the collection of tissue samples, which were weighed, dissolved in 7 M potassium hydroxide and counted in a gamma counter (LKB model 1270). Tissue uptake was calculated as the mean percentage of the injected dose per gram of tissue (% ID $g^{-1}$) from a group of four mice.

### Results

*Preparation of immunoconjugates and radiolabelling*

For correct interpretation of *in vivo* data it is important to characterise the immunoconjugates prepared. The number of chelation groups for $^{111}$In was controlled at 0.5–1.0 in each case to minimise any possible damage to the antibody as modification with high levels of cDTPA or 2-iminothiolane is known to cause loss of immunoreactivity and often aggregation of the antibody (Paik *et al.*, 1985; Sakahara *et al.*, 1985). For the macrocycle and the CT-DTPA conjugates, this was achieved by reacting a small aliquot of the antibody with 4,4'-dithiodipyridine (DTDP) immediately after 2-iminothiolane modification to determine the number of generated thiol groups and then again on another aliquot after reaction with the appropriate maleimide linker to determine how many thiol groups were remaining. The difference in the number of titratable thiols in the two assays gave the number of chelates per antibody. Controls were carried out to show that in the absence of the linkers the loss of thiols by oxidation was minimal. For the cDTPA conjugate the number of chelates per antibody was determined by a radiolabelling HPLC/ITLC assay (Meares *et al.*, 1984).

Preparation of conjugates with CT-DTPA, 9N3 and 12N4 resulted in only very low levels of aggregation visible on HPLC (less than 5% in each case). SDS-PAGE analysis of these conjugates also showed no difference in the banding pattern to the unmodified antibody when examined under non-reducing conditions on an 8% gel or reducing conditions on a 12% gel. HPLC analysis of the cDTPA conjugate also revealed less than 5% aggregate present by HPLC, but in this case some high molecular weight forms were noticed by SDS-PAGE, suggesting that more damage was caused to the antibody during the preparation of this conjugate. The antigen-binding ability of the immunoconjugates was unchanged from the unmodified antibody, probably as a result of keeping the number of chelation groups attached to a minimum ($>0.5$, $<1.0$). Further purification of the immunoconjugates was not carried out at this stage as the small amounts of aggregate present were removed by HPLC after radiolabelling.

Stability of the labelled preparations was considered to be very important, and as a result stripping experiments using DTPA were initially performed on both the free maleimide derivatives of 9N3 and 12N4 as well as the immunoconjugates. The derivatives were labelled with $^{111}$In and purified by injection onto HPLC ion exchange in series with a radiochemical detector and eluted isocratically with 70% water–20% 1 M ammonium acetate, pH 6.5–10% acetronitrile. The labelled linkers were then incubated in 5 mM DTPA for up to 24 h at room temperature, and the loss of the $^{111}$In from the macrocycle was monitored by the increase in the $^{111}$In-DTPA peak on HPLC. There was a loss of $<3\%$ of $^{111}$In from the 9N3 linker, whereas there was $>40\%$ loss from the 12N4 linker over the same time period.

A similar phenomenon to that seen with the free macrocycle derivatives was observed with the $^{111}$In-labelled 9N3 and 12N4 immunoconjugates when the HPLC-purified samples (in 0.2 M sodium phosphate–5 mM DTPA, pH 7.0) were assessed for radiochemical purity. One IgG-associated peak for the 9N3 conjugate was seen, whereas two peaks were seen for the 12N4 conjugate found to be IgG and low molecular weight material ($^{111}$In-DTPA), the latter accounting for 25% of the total counts. Figure 2 shows the HPLC/radiochemical detector profiles obtained at various stages in the preparation of the final immunoconjugate. Hence, the 12N4 conjugate was less stable, with 25% of the activity dissociating from the antibody after overnight incubation. Removal of the $^{111}$In-DTPA from the 12N4 conjugate preparation by G-25 Sephadex chromatography resulted in a stable conjugate with no further loss of $^{111}$In. The incubation was continued for a further period of 3 days, but no further loss of $^{111}$indium was seen.

Experiments were also performed in which non-thiolated cB72.3 was incubated with the maleimide derivative of both the 9N3 and 12N4 linkers and then labelled with $^{111}$In in the same way. HPLC/radiochemical detector analysis showed that the counts were only associated with low molecular weight material, that is $^{111}$In-12N4 and $^{111}$In-DTPA (data not shown). This meant that there was no non-specific attachment of the chelates to the antibody.

These data suggested that the 12N4 macrocycle binds $^{111}$In in two forms, one of which is loosely bound and can be stripped from the conjugate by incubation in DTPA and the other of which is apparently stable. Indium-111 binding by the 12N4 macrocycle has been examined by $^{13}$C nuclear magnetic resonance (NMR) studies in both acetate and succinate buffers (pH 5–6), but only one complex was seen (D. Parker, unpublished data). In view of these results, the labelling procedure for the 12N4 conjugate was modified to include an overnight DTPA stripping step prior to purification for *in vivo* use. Similar stripping studies with the CT-DTPA and cDTPA immunoconjugates revealed that all of the activity was IgG bound. The final radiolabelling efficiencies are shown in Table I. The initial uptake of $^{111}$In by the 12N4 macrocycle conjugate (~60%) was lower than for the 9N3,

**Figure 2** HPLC/radiochemical detector profiles of $^{111}$In-labelled cB72.3-9N3 and cB72.3-12N4 conjugates and before and after HPLC purification, illustrating the requirement of the DTPA stripping step for the 12N4 conjugate. **a**, $^{111}$In-DTPA at 12.22 min; **b**, $^{111}$In-cB72.3-9N3 before HPLC purification, at 7.59 min with $<5\%$ aggregate peak; **c**, $^{111}$In-cB72.3-12N4 before HPLC purification, at 7.59 min with a free $^{111}$In-DTPA peak at 12.22 min; **d**, $^{111}$In-cB72.3-9N3 after HPLC purification, at 8.31 min showing removal of the aggregate peak and free $^{111}$In-DTPA; **e**, $^{111}$In-cB72.3-12N4 after HPLC purification followed by incubation in 5 mM DTPA, pH 7.0, overnight; **f**, $^{111}$In-cB72.3-12N4 prepared as in **e** after buffer exchange into PBS to remove $^{111}$In-DTPA prior to injection into mice.

cDTPA and CT-DTPA chelates and dropped further to 35% after the stripping step.

All labelled immunoconjugates were purified by preparative HPLC before use for *in vivo* studies so that the small amounts of aggregate and free indium present were removed, allowing the preparation of very high-quality labelled immunoconjugates. The resulting preparations contained no detectable aggregate or free $^{111}$In by duplicate HPLC (Figure 2) and ITLC assays. SDS-PAGE/autoradiography of the labelled preparations also revealed a high degree of purity. The bands on the autoradiograph corresponded to the Coomassie blue-stained bands on SDS-PAGE.

### *Biodistribution in nude mice bearing LS174T xenografts*

The tissue distributions of cDTPA, CT-DTPA and 9N3 conjugates were compared at 24, 48 and 144 h. The results show

**Table I** Radiolabelling efficiency of immunoconjugates

| *Immunoconjugate* | *Radiolabelling efficiency (final) (%)* |
|---|---|
| cB72.3-cDTPA | 86 |
| cB72.3-CT-DTPA | 90 |
| cB72.3-9N3 | 95 |
| cB72.3-12N4 | 35 |

Radiolabelling was carried out as described in the text and the efficiency determined by HPLC/radiochemical detector. For cDTPA, CT-DTPA and 9N3 conjugates radiolabelling was performed in 0.1 M sodium acetate, pH 5.0, at 20°C for 15 min, quenched by the addition of 5 mM DTPA and HPLC purified. For the 12N4 conjugate radiolabelling was carried out as above except that an overnight stripping step in 5 mM DTPA was included after the HPLC purification.

**Table II** Comparative biodistribution of $^{111}$In-labelled cB72.3 conjugates in LS-174T tumour-bearing mice

| *Tissue* | *9N3* | *cDTPA* | *CT-DTPA* |
|---|---|---|---|
| 24 h | | | |
| Blood | 13.33 (0.62) | 9.34 (0.68) | 15.02 (1.43) |
| Liver | 4.40 (0.38) | 4.95 (0.67) | 4.83 (0.65) |
| Kidney | 13.30 (0.80) | 10.32 (0.71) | 18.57 (0.43) |
| Lung | 5.96 (0.19) | 4.70 (0.73) | 6.52 (0.63) |
| Spleen | 3.30 (0.18) | 3.31 (0.38) | 3.44 (0.21) |
| Colon | 1.61 (0.06) | 1.53 (0.14) | 1.61 (0.11) |
| Muscle | 1.49 (0.13) | 1.73 (0.31) | 1.34 (0.15) |
| Femur | 1.61 (0.06) | 1.49 (0.17) | 1.83 (0.15) |
| Tumour | 14.48 (1.48) | 10.75 (0.52) | 13.44 (1.82) |
| 48 h | | | |
| Blood | 12.87 (2.12) | 8.39 (0.57) | 14.16 (1.82) |
| Liver | 3.94 (0.47) | 5.79 (0.29) | 4.77 (0.30) |
| Kidney | 17.39 (1.23) | 14.20 (0.79) | 35.54 (1.87) |
| Lung | 5.58 (0.97) | 4.37 (0.24) | 6.45 (0.28) |
| Spleen | 3.49 (0.42) | 3.85 (0.13) | 3.77 (0.05) |
| Colon | 1.35 (0.32) | 2.23 (0.11) | 1.66 (0.09) |
| Muscle | 1.17 (0.20) | 1.40 (0.17) | 1.39 (0.21) |
| Femur | 1.68 (0.21) | 2.63 (0.16) | 2.01 (0.14) |
| Tumour | 22.88 (1.76) | 12.79 (1.56) | 18.20 (0.94) |
| 144 h | | | |
| Blood | 3.87 (2.12) | 2.99 (0.67) | 6.78 (0.67) |
| Liver | 3.18 (0.47) | 5.81 (0.20) | 3.27 (0.21) |
| Kidney | 10.68 (1.23) | 10.63 (1.17) | 22.66 (2.44) |
| Lung | 2.26 (0.97) | 3.03 (0.31) | 3.50 (0.25) |
| Spleen | 2.63 (0.42) | 3.76 (0.37) | 3.77 (0.36) |
| Colon | 0.61 (0.32) | 1.24 (0.11) | 3.77 (0.36) |
| Muscle | 0.85 (0.20) | 1.45 (0.36) | 0.82 (0.04) |
| Femur | 1.00 (0.21) | 1.94 (0.43) | 1.28 (0.09) |
| Tumour | 14.45 (1.76) | 8.33 (0.90) | 15.50 (2.60) |

Results expressed as the mean per cent injected dose per gram of tissue (standard error, $n = 4$).

**Figure 3** Graphical representation of tissue-to-blood ratios of $^{111}$In-labelled cB72.3 conjugates in LS-174T tumour-bearing mice: **a**, liver; **b**, femur; **c**, colon; **d**, spleen for 9N3 (from Table II). ( ▆ ), 9N3 (from Table III) ( ▨ ), cDTPA; ( ▩ ), CT-DTPA ( ▧ ) and 12N4 ( ☐ ). In each case the ratios calculated from individual mouse data are plotted as mean per cent injected dose per gram of tissue divided by mean per cent injected dose of blood. Error bars indicate standard errors of the mean.

(Table II) that the tumour uptake was significantly higher for the 9N3 conjugate at each time point ($P<0.05$) compared with the cDTPA conjugate, but similar to the CT-DTPA conjugate. This was a result of the significantly lower blood levels ($P<0.05$) for the cDTPA conjugate. The liver levels were low for all three conjugates but differed significantly for cDTPA at 144 h ($P<0.01$). Another significant difference in biodistribution was seen in the femur, where the levels were higher for the cDTPA conjugate ($P<0.05$). Levels of activity in the kidney were relatively high and variable with this chimeric antibody for all chelates, and especially for CT-DTPA. This is known to be a particular property in four known cases (three unpublished) of human IgG4 antibodies resulting from the proportion of human IgG4 which does not fully form its hinge disulphide bonds (Angal *et al.*, 1993, and unpublished data). Overall the most favourable distribution was observed for the 9N3 conjugate.

A further biodistribution study was then undertaken to compare the tissue distribution of $^{111}$In-labelled 9N3 macrocycle conjugate with a 12N4 macrocycle conjugate labelled with $^{111}$In and stripped with DTPA as described. The results at 24, 96 and 144 h are shown in Table III. In this experiment the difference between the two conjugates was not so pronounced. However, the differences were mainly seen at 144 h, when the liver and the femur levels were significantly higher for the 12N4 conjugate ($P<0.05$).

To allow comparison of tissues without the contribution of differences in blood-associated activity, the tissue-to-blood ratios were calculated (% ID $g^{-1}$ tissue divided by % ID $g^{-1}$ blood). The ratios were obtained by calculating the means (and standard error) of the ratios of individual mice in each group. These ratios emphasised the difference between the chelates, especially with respect to the spleen, liver, colon and femur (Figure 3), instability of the cDTPA conjugate being seen especially at the longer time points. Some variation was observed in the blood levels between the different cB72.3-9N3 chelates used in the two experiments, however this was largely due to the variation within groups of mice. When the tissue-to-blood ratios were examined it was evident that there was no real difference.

The tumour accumulation was directly related to the blood clearance as evidenced by the tumour-to-blood ratios (calculated from individual mouse data), which were similar for all of the chelates. There was some evidence of higher tumour-to-tissue ratios for the 9N3 conjugate compared with the 12N4 conjugate in the liver, colon and femur, especially at the longer time point (144 h, Figure 4).

**Table III** Comparative biodistribution of $^{111}$In-labelled cB72.3 macrocyclic conjugates in LS-174T tumour-bearing mice

| *Tissue* | *9N3* | *12N4* |
|---|---|---|
| 24 h | | |
| Blood | 9.84 (0.81) | 13.24 (2.34) |
| Liver | 3.82 (0.54) | 4.47 (0.50) |
| Kidney | 11.99 (1.25) | 16.86 (1.51) |
| Lung | 4.73 (0.71) | 5.27 (0.80) |
| Spleen | 2.64 (0.28) | 3.23 (0.25) |
| Colon | 1.38 (0.18) | 1.96 (0.21) |
| Muscle | 1.21 (0.12) | 1.89 (0.19) |
| Femur | 1.43 (0.22) | 2.17 (0.31) |
| Tumour | 10.36 (1.01) | 9.59 (0.53) |
| 96 h | | |
| Blood | 7.28 (0.35) | 6.30 (0.91) |
| Liver | 3.70 (0.20) | 6.40 (0.63) |
| Kidney | 15.29 (1.93) | 15.14 (1.75) |
| Lung | 3.71 (0.38) | 4.14 (0.77) |
| Spleen | 3.11 (0.42) | 4.48 (0.60) |
| Colon | 1.05 (0.09) | 1.46 (0.14) |
| Muscle | 1.00 (0.26) | 0.84 (0.21) |
| Femur | 1.21 (0.09) | 1.94 (0.32) |
| Tumour | 12.72 (1.54) | 10.44 (1.32) |
| 144 h | | |
| Blood | 4.85 (0.73) | 4.83 (0.44) |
| Liver | 3.62 (0.19) | 5.41 (0.65) |
| Kidney | 12.76 (1.90) | 14.62 (1.30) |
| Lung | 3.22 (0.46) | 3.06 (0.19) |
| Spleen | 3.08 (0.32) | 3.94 (0.22) |
| Colon | 0.89 (0.08) | 1.45 (0.27) |
| Muscle | 0.94 (0.16) | 1.33 (0.09) |
| Femur | 0.99 (0.15) | 1.59 (0.25) |
| Tumour | 11.31 (1.03) | 9.69 (0.78) |

Results expressed as mean per cent injected dose per gram of tissue (standard error, $n = 4$).

### Discussion

The aim of this work was to identify the best method for the attachment of $^{111}$In to antibodies for tumour imaging purposes and to compare with the relevant clinical standard, cDTPA. To achieve this, several factors need to be taken into account, including the ease and speed of preparation of the labelled antibody, the radiolabelling efficiency and the *in vivo* biodistribution. We have shown that all of the conjugates prepared here are capable of being labelled with $^{111}$In, with both the bifunctional DTPA derivative CT-DTPA and 9N3

**Figure 4** Graphical representation of tumour to tissue ratios of $^{111}$In-labelled cB72.3 conjugates in LS-174T tumour-bearing mice at **a**, 24 h and **b**, 144 h for 9N3 (from Table II), ( ■ ), 9N3 (from Table III) ( ▨ ), cDTPA ( ▩ ), CT-DTPA ( ▧ ) and 12N4 ( □ ). In each case the ratios calcuated from individual mouse data are plotted as the mean per cent injected dose per gram of tumour divided by the mean per cent injected dose per gram of tissue. Error bars indicate standard errors of the mean.

macrocycle labelling to particularly high efficiency. The 12N4 macrocycle is somewhat more difficult to use with $^{111}In$ because of the need for a DTPA stripping step to remove loosely bound $^{111}In$.

Great care was taken in the preparation of labelled conjugates for *in vivo* studies. They were prepared by protocols designed to minimise antibody damage and purified by HPLC to produce high-quality labelled conjugates. This has been reported to improve biodistribution and consequently scintigraphic images (Esteban *et al.*, 1987). Indeed, as a result our own biodistribution data with the cDTPA conjugate were seemingly better than published data obtained with the murine version of the B72.3 antibody (Brown *et al.*, 1987; Esteban *et al.*, 1987).

The $^{111}In$-cB72.3-9N3 conjugate was shown to be more stable *in vivo* than cDTPA, as evidenced by significantly lower liver, femur and colon uptake. This was particularly emphasised by the tissue-to-blood ratios, which increased with time for the cDTPA conjugate as $^{111}In$ was lost from the blood and accumulated in a range of tissues. The bifunctional DTPA derivative, CT-DTPA, also showed an improved biodistribution over cDTPA, as expected by comparison with other substituted DTPA chelates (Roselli *et al.*, 1989). Comparison of CT-DTPA and the 9N3 macrocycle biodistributions showed them to be very similar, with the major difference being the lower kidney levels for 9N3. Both CT-DTPA and the 9N3 macrocycle are attractive as chelators for $^{111}In$, with 9N3 being preferable.

The biodistribution of the 12N4 macrocycle conjugate showed poorer tumour-to-tissue ratios than the 9N3 macrocycle conjugate, however the data showed that both of these macrocycles are capable of $^{111}In$ chelation in a stable form. The attraction of using the 12N4 macrocycle for $^{111}In$ is that this macrocycle can also chelate $^{90}Y$ in a very stable form with improved biodistribution over cDTPA and CT-DTPA (Meares *et al.*, 1990; Harrison *et al.*, 1991). This would allow the use of the same antibody conjugate for both imaging with $^{111}In$ and therapy with $^{90}Y$. However, the more difficult and time-consuming labelling procedure to achieve stable $^{111}In$ uptake by 12N4 makes this alternative less attractive. The importance of this procedure is emphasised by the work of Snook *et al.* (1991), who found that high liver levels of activity were seen (similar to those seen with cDTPA) when a similar derivative of 12N4 was tested without a DTPA stripping step in the labelling procedure.

Some preliminary clinical studies have been reported using a similar 12N4 macrocycle to that used in this study, attached to the antibodies HMFG1 and a humanised version of H17E2 (Hird *et al.*, 1991; Kosmas *et al.*, 1992). In these studies an immune response was generated by the patients to the macrocycle, which appeared to act as a hapten when attached to the antibody. The macrocyclic ligand used was linked to the antibody through a benzyl-containing structure, and is therefore a very different type of structure to those used here, in which no aromatic rings are present. In addition, high levels of aggregate (up to 16%) were present in the preparations used by Kosmas *et al.* (1992). However, the immunogenicity of the 9N3 and 12N4 macrocyclic conjugates used in this report remains to be evaluated.

Overall, the malemide derivative of the 9N3 macrocycle is an ideal reagent for the attachment of $^{111}In$ to antibodies in a stable form, it can be attached in a simple procedure without causing antibody damage, can be labelled to high efficiency and gives rise to a very stable conjugate in biodistribution studies. Indeed, a preliminary report of clinical data with this reagent attached to the antibody SM3 has revealed superior tumour imaging to that obtained with the same antibody labelled with DTPA (Granowska *et al.*, 1991). The combination of stable attachment methods for radioisotopes such as the macrocyclic ligands reported here with recombinant humanised antibodies, which may allow repeat dosing through reduction of the immune response (Adair, 1992), should allow the development of a new generation of radiolabelled antibody conjugates for tumour imaging and therapy.

We would like to thank Dr Dan Shochat of American Cyanamid, Pearl River, New York, for helpful discussions and advice. We are also grateful to Tina Jones for assistance in the preparation of figures.

**Abbreviations:** cDTPA, cyclic diethylenetriaminepentaacetic acid anhydride; % ID $g^{-1}$, mean percentage injected dose per gram; CT-DTPA, C-linked derivative of DTPA; 9N3, c-functionalised derivative of 1,4,7-triazacyclonanetriacetic acid; 12N4, C-functionalised derivative of 1,4,7,10-tetraazacyclododecanetetraacetic acid; RI, radiolocalisation indices (percentage injected dose per gram tumour divided by percentage injected dose per gram tissue); ITLC, instant thin-layer chromatography; DTDP, 4,4'-dithiodipyridine; DMSO, dimethylsulphoxide.

### References

ADAIR, J.R. (1992). Engineering antibodies for therapy. *Immunol. Rev.*, **130**, 5-40.

ANGAL, S., KING, D.J., BODMER, M.W., TURNER, A., LAWSON, A.D.G., ROBERTS, G., PEDLEY, B. & ADAIR, J.R. (1993). A single amino acid substitution abolishes the heterogeneity of chimeric mouse/human IgG4 antibody. *Mol. Immunol.*, **30**, 105-108.

BEGENT, R.H.J., LEDERMANN, J.A., BAGSHAWE, K.D., GREEN, A.J., KELLY, A.M.B., LANE, D., SECHER, D.S., DEWJI, M.R. & BAKER, T.S. (1990). Phase I/II study of chimeric B72.3 antibody in radioimmunotherapy of colorectal carcinoma. *Br. J. Cancer*, **62**, 487.

BRECHBIEL, M.W., GANSOW, O.A., ATCHER, R.W., SCHLOM, J., ESTEBAN, J., SIMPSON, D. & COLCHER, D. (1986). Synthesis of 1-(*p*-isothiocyanatobenzyl) derivatives of DTPA and EDTA. Antibody labeling and tumour imaging studies. *Inorg. Chem.*, **25**, 2772-2781.

BROAN, C.J., COX, J.P.L., CRAIG, A.S., KATAKY, R., PARKER, D., HARRISON, A., RANDALL, A.M. & FERGUSON, G. (1991). Structure and solution stability of indium and gallium complexes of 1,4,7-triazacyclononanetriacetate and yttrium complexes of 1,4,7,10-tetraazacyclododecanetetraacetate and related ligands. *J. Chem. Soc. Perkin Trans.*, **2**, 87-99.

BROWN, B.A., COMEAU, R.D., JONES, P.L., LIBERATORE, F.A., NEACY, W.P., SANDS, H. & GALLAGHER, B.M. (1987). Pharmacokinetics of the monoclonal antibody B72.3 and its fragments labelled with either $^{125}I$ or $^{111}In$. *Cancer Res.*, **47**, 1149-1154.

COLCHER, D., KEENAN, A.M., LARSON, S.M. & SCHLOM, J. (1986). Prolonged binding of a radiolabelled monoclonal antibody (B72.3) used for the *in situ* radioimmunodetection of human colon carcinoma xenografts. *Cancer Res.*, **44**, 5744-5751.

COLCHER, D., MILENIC, D., ROSELLI, M., RAUBITSCHEK, A., YARRANTON, G., KING, D., ADAIR, J., WHITTLE, N., BODMER, M. & SCHLOM, J. (1989). Characterisation and biodistribution of recombinant and recombinant/chimeric constructs of monoclonal antibody B72.3. *Cancer Res.*, **49**, 1738-1745.

COX, J.P.L., JANKOWSKI, K.J., KATAKY, R., PARKER, D., BEELEY, N.R.A., BOYCE, B.A., EATON, M.A.W., MILLAR, K., MILLICAN, T.A., HARRISON, A. & WALKER, C. (1989). Synthesis of a kinetically stable yttrium-90 labelled macrocycle antibody conjugate. *J. Chem. Soc., Chem. Commun.*, **12**, 797-798.

CRAIG, A.S., HELPS, I.A., JANOWSKI, K.J., PARKER, D., BEELEY, N.R.A., BOYCE, B.A., EATON, M.A.W., MILLICAN, T.A., MILLAR, K., PHIPPS, A., RHIND, S.K., HARRISON, A. & WALKER, C. (1989). Towards tumour imaging with Indium-111 labelled macrocycle antibody conjugates. *J. Chem. Soc., Chem. Commun.*, **12**, 794-796.

DAVIDSON, B.R., BABICH, J., YOUNG, H., WADDINGTON, W., CLARKE, G., SHORT, M., BOULOS, P., STYLES, J. & DEAN, C. (1991). The effect of circulating antigen and radiolabel stability on the biodistribution of an indium labelled antibody. *Br. J. Cancer*, **64**, 850-856.

DESHPANDE, S.V., SUBRAMANIAN, R., MCCALL, M.J., DENARDO, S.J., DENARDO, G.L. & MEARES, C. (1990). Metabolism of Indium chelates attached to monoclonal antibody: minimal transchelation of Indium from benzyl-EDTA chelate *in vivo*. *J. Nucl. Med.*, **31**, 218–224.

DIVGI, C.R., MCDERMOTT, K., JOHNSON, D.K., SCHNOBRICH, K.E., FINN, R.D., COHEN, A.M. & LARSON, S.M. (1991). Detection of hepatic metastases from colorectal carcinoma using indium-111 ($^{111}$In) labeled monoclonal antibody (mAb): MSKCC experience with mAb $^{111}$In-C110. *Nucl. Med. Biol.*, **18**, 705–710.

ESTEBAN, J.M., SCHLOM, J., GANSOW, O.A., ATCHER, R.W., BRECHBIEL, M.W., SIMPSON, D.E. & COLCHER, D. (1987). New method for the chelation of $^{111}$In to monoclonal antibodies: biodistribution and imaging of athymic mice bearing human colon carcinoma xenografts. *J. Nucl. Med.*, **28**, 861–870.

GOLDENBERG, D.M., WLODKOWSKI, T.J., SHARKEY, R.M., SILBERSTEIN, E.B., SERAFINI, A.N., GARTY, I.I., VAN HEERTUM, R.L., HIGGINBOTHAM-FORD, E.A., KOTLER, J.A., BALASUBRIAMANIAN, N., SWAYNE, L.C., HANSEN, H.J. & PINSKY, C.M. (1993). Colorectal cancer imaging with iodine-123 labelled CEA monoclonal antibody fragments. *J. Nucl. Med.*, **34**, 61–70.

GRANOWSKA, M., MATHER, S.J. & BRITTON, K.E. (1991). Diagnostic evaluation of $^{111}$In and $^{99m}$Tc radiolabelled monoclonal antibodies in ovarian and colorectal cancer: correlations with surgery. *Nucl. Med. Biol.*, **18**, 413–424.

HARRISON, A., WALKER, C.A., PARKER, D., JANKOWSKI, K.J., COX, J.P.L., CRAIG, A.S., SANSOM, J.M., BEELEY, N.R.A., BOYCE, R.A., CHAPLIN, L., EATON, M.A.W., FARNSWORTH, A.P.H., MILLAR, K., MILLICAN, A.T., RANDALL, A.M., RHIND, S.K., SECHER, D.S. & TURNER, A. (1991). The *in vivo* release of $^{90}$Y from cyclic and acyclic ligand antibody conjugates. *Nucl. Med. Biol.*, **18**, 469–476.

HIRD, V., VERHOEYEN, M., BADLEY, R.A., PRICE, D., SNOOK, D., KOSMAS, C., GOODEN, C., BAMIAS, A., MEARES, C., LAVENDER, J.P. & EPPENETOS, A.A. (1991). Tumour localisation with a radioactively labelled reshaped human monoclonal antibody. *Br. J. Cancer*, **64**, 911–914.

HNATOWICH, D.J., CHILDS, R.L., LANTEIGNE, D. & NAJAFI, A. (1983). The preparation of DTPA coupled antibodies radiolabelled with metallic radionuclides: an improved method. *J. Immunol. Methods*, **65**, 147–157.

HNATOWICH, D.J., GRIFFEN, T.W., KOSCIUCZYK, C., RUSCKOWSKI, M., CHILDS, R.L., MATTIS, J.A., SHEALY, D. & DOCHERTY, P.W. (1985). Pharmacokinetics of an $^{111}$In labelled monoclonal antibody in cancer patients. *J. Nucl. Med.*, **26**, 849–858.

KHAZAELI, M.B., SALEH, M.N., LIU, T.P., MEREDITH, R.F., WHEELER, R.H., BAKER, T.S., KING, D., SECHER, D., ALLEN, L., ROGERS, K., COLCHER, D., SCHLOM, J., SHOCHAT, D. & LOBUGLIO, A.F. (1991). Pharmacokinetics and immune response of $^{131}$I chimeric mouse/human B72.3 (human gamma 4) monoclonal antibody in man. *Cancer Res.*, **51**, 5461–5466.

KING, D.J., MOUNTAIN, A., ADAIR, J.R., OWENS, R.J., HARVEY, A., WEIR, N., PROUDFOOT, K.A., PHIPPS, A., LAWSON, A., RHIND, S.K., PEDLEY, B., BODEN, J., BODEN, R., BEGENT, R.H.J. & YARRANTON, G.T. (1992). Tumour localization of engineered antibody fragments. *Antibody Immunoconj. Radiopharm.*, **5**, 159–170.

KOSMAS, K., SNOOK, D., GOODEN, C.S., COURTENAY-LUCK, N.J., MCCALL, M.J., MEARES, C. & EPPENETOS, A.A. (1992). Development of humoral immune responses against a macrocyclic chelating agent (DOTA) in cancer patients receiving radioimmunoconjugates for imaging and therapy. *Cancer Res.*, **52**, 904–911.

LAMKI, L.M., BUZDAR, A.U., SINGLETARY, E., ROSENBLUM, M.G., BHADKAMKAR, V., ESPARZA, L., PODOLOFF, D.A., ZUKIWSKI, A., HORTOBAGYI, G.N. & MURRAY, J.L. (1991). Indium-111 labelled B72.3 monoclonal antibody in the detection and staging of breast cancer: a phase I study. *J. Nucl. Med.*, **32**, 1326–1332.

LYONS, A., KING, D.J., OWENS, R.J., YARRANTON, G.T., MILLICAN, T.A., WHITTLE, N.R. & ADAIR, J.R. (1990). Site specific attachment to recombinant antibodies via introduced surface cysteine residues. *Protein Eng.*, **3**, 703–708.

LUNDY, J., GREINER, J.W. & COCHER, D. (1986). Development of a metastatic human colon cancer xenograft model in the nude mouse. *J. Surg. Oncol.*, **31**, 260–267.

MEARES, C.F., MCCALL, M.J., REARDON, D.T., GOODWIN, D.A., DIAMANTI, C.I. & MCTIGUE, M. (1984). Conjugation of antibodies with bifunctional chelating agents: isothiocyanate and bromoacetamide reagents, methods of analysis and subsequent addition of metal ions. *Anal. Biochem.*, **142**, 68–78.

MEARES, C.F., MOI, M.K., DIRIL, H., KUKIS, D.L., MCCALL, M.J., DESHPANDE, S.V., DENARDO, S.J., SNOOK, D. & EPENETOS, A.A. (1990). Macrocyclic chelates of radiometals for diagnosis and therapy. *Br. J. Cancer*, **62** (Suppl. X), 21–26.

MOI, M.K., MEARES, C.F. & DENARDO, S.J. (1988). The peptide way of macrocyclic bifunctional chelating agents. Synthesis of 2-(p-nitrobenzyl)-1,4,7,10-teracyclododecane-N,N',N'',N''',N''''-tetraacetic acid, and study of its Yttrium (III) complex. *J. Am. Chem. Soc.*, **110**, 6266.

PAIK, C.H., HONG, J.J., EBBERT, M.A., HEALD, S.C., REBA, C. & ECKELMAN, W.C. (1985). Relative reactivity of DTPA, immunoreactive antibody-DTPA conjugates and non-immunoreactive antibody-DTPA conjugates toward $^{111}$In. *J. Nucl. Med.*, **26**, 482–487.

ROSELLI, M., SCHLOM, J., GANSOW, O.A., RAUBITSCHEK, A., MIRZADEH, S., BRECHBIEL, M.W. & COLCHER, D. (1989). Comparative biodistributions of yttrium and indium labeled monoclonal antibody B72.3 in athymic mice bearing human colon carcinoma xenografts. *J. Nucl. Med.* **30**, 672-682.

SAKAHARA, H., ENDO, K., NAKASHIMA, T., KOIZUMI, M., OHTA, H., TORIZUKA, K., FURUKAWA, T., OHMOMO, Y., YOKOYAMA, A., OKADA, K., YOSHIDA, O. & NISHI, S. (1985). Effect of DTPA conjugation on the antigen binding activity and biodistribution of monoclonal antibodies against alpha-feto-protein. *J. Nucl. Med.*, **26**, 750–755.

SCHUMAKER, J., KLIVENYI, G., MATYS, R., KIRCHGEBNER, H., HAUSER, H., MAIER-BORST, W. & MATZU, S. (1990). Uptake of indium-111 in the liver of mice following administration of indium-111 DTPA-labeled monoclonal antibodies: influence of labeling parameters, physiologic parameters and antibody dose. *J. Nucl. Med.*, **31**, 1084–1093.

SNOOK, D.E., ROWLINSON-BUSZA, G., MEARES, C. & EPENETOS, A.A. (1991). Indium-111 and yttrium-90 labelled macrocyclic chelating agents. In *Monoclonal Antibodies*, Epenetos A.A. (ed.) pp. 157–166. Chapman & Hall: London.

WHITTLE, N., ADAIR, J., LLOYD, C., JENKINS, L., DEVINE, J., SCHLOM, J., RAUBITSCHEK, A., COLCHER, D. & BODMER, M. (1987). Expression in COS cells of a mouse–human chimaeric B72.3 antibody. *Protein Eng.*, **1**, 499–505.